# Efficient Multi-Sound Source Localization Algorithm for Transformer Faults Based on Polyphase Filters

**DOI:** 10.3390/s24020604

**Published:** 2024-01-17

**Authors:** Hualiang Zhou, Zhantao Su, Yuxuan Huang, Lu Lu, Mingwei Shen

**Affiliations:** 1 NARI Group Corporation (State Grid Electric Power Research Institute), Nanjing 211106, China; suzhantao@sgepri.sgcc.com.cn (Z.S.); lulu1@sgepri.sgcc.com.cn (L.L.); 2 NARI Technology Nanjing Control System Co., Ltd., Nanjing 211106, China; 3 College of Information Science and Engineering, Hohai University, Nanjing 210098, China; yx_huang@hhu.edu.cn (Y.H.); smw_nuaa@hhu.edu.cn (M.S.)

**Keywords:** transformer, polyphase filter, sum-difference monopulse, multi-source separation, multi-source localization

## Abstract

Power transformers play a critical role in power systems, and the early detection of their faults and defects, accounting for over 30%, can be achieved through abnormal sound analysis. Sound source localization based on microphone arrays has proven effective in focusing on the troubleshooting scope, preventing potential severe hazards caused by delays in fault removal, and significantly reducing operational and maintenance difficulties and costs. However, existing microphone array-based sound source localization algorithms face challenges in maintaining both accuracy and simplicity and especially suffer from a sharp decrease in performance when dealing with multiple sound sources. This paper presents a multi-sound source localization algorithm for transformer faults based on polyphase filters, integrating the sum-difference monopulse angle measurement technique into the microphone array. Firstly, the signals received from the transformers are divided into multiple subbands using polyphase filters, allowing for multi-source separation and reducing the sampling rate of each subband. Next, the time-domain signals in subbands subject to noise suppression are processed into sum and difference beams. The resulting beam outputs are transformed into frequency-domain signals using the Fast Fourier Transform (FFT), effectively enhancing the signal-to-noise ratio (SNR) for separate sound sources. Finally, each subband undergoes sum-difference monopulse angle measurement in the frequency domain to achieve the high-precision localization of specific faults. The proposed algorithm has been demonstrated to be effective in achieving higher localization accuracy and reducing computational complexity in the presence of actual amplitude-phase errors in microphone arrays. These advantages can facilitate its practical applications. By enabling early targeting of fault sources when abnormalities occur, this algorithm provides valuable assistance to operation and maintenance personnel, thereby enhancing the maintenance efficiency of transformers.

## 1. Introduction

The rapid growth in power demand has led to the constant expansion of grid capacity and the continuous improvement of voltage levels. As a result, higher and stricter requirements for safe operation and reliable power supply have been imposed on power systems. Power transformers serve as core equipment in power systems, and their operating conditions have a direct impact on the overall functioning of power grids [1,2,3]. It is essential to enhance the operational reliability of transformers as well as promptly identify and localize any faults that occur during their operation, which is a crucial task for ensuring the safe and reliable operation of power grids. At present, some typical faults of power transformers, such as partial discharge, short-circuit impulse, and DC magnetic bias, can be distinguished from the voiceprint signal. For example, when a partial discharge occurs in a power transformer, air bubbles and impurities will appear inside, leading to partial damage to the dielectric and a “squeak” or “crackle” sound. When a power transformer encounters a short-circuit shock, the short-circuit current will surge, producing a “gurgling” boiling sound inside the transformer [4]. Therefore, in engineering design, when facing voiceprint information, it is necessary to first detect and identify the faulty voiceprint, and then locate the identified faulty voiceprint. However, due to various factors such as the presence of interference sounds in their operating environments, complex structures, and diverse operating conditions, existing localization techniques can only target large components, falling short of achieving precise localization. In recent years, microphone array-based sound source localization technology has gained significant attention due to its convenience and efficiency [5,6,7,8,9].

Many scholars, both in China and abroad, have conducted extensive studies on microphone array-based sound source localization. For instance, Hahn et al. proposed a sound source localization method based on steerable beamforming [10]. However, this method relies on prior knowledge of sound sources and ambient noises, which is often challenging to obtain in real-world scenarios. Another method presented by Dibiase et al. is the guided response power-phase conversion approach [11]. However, this technique necessitates a global search and a subsequent heavy computational workload. Schmidt introduced the multiple signal classification (MUSIC) algorithm [12], which is only applicable in scenarios where the number of sources is known and the sources are non-coherent. This algorithm has subsequently sparked various improvements by other researchers. For instance, Reference [13] suggests using a windowing function to filter and process coherent signals for estimating their direction of arrival (DOA). Reference [14] proposes a joint diagonalization matrix-based algorithm to construct a cost function, enabling DOA estimation even in scenarios with unknown numbers of mixed signals. Although these improved versions of the MUSIC algorithm have made progress in overcoming the limitations of the original method, they still face challenges in real-time processing due to the high computational complexity associated with covariance matrix estimation and matrix inversion.

While these algorithms demonstrate effectiveness in localizing sound sources under specific scenarios, applying them directly in complex multi-source transformer environments is impractical. In such scenarios, a feasible approach to localizing each sound source involves using a filter bank for separate extraction of sources, followed by direction finding. However, traditional analysis filter banks perform filtering before sampling, which can lead to increased hardware resource requirements when a high sampling rate is necessary. In contrast, the utilization of polyphase filters (PFs) [15,16,17] offers advantages in improving resource efficiency by sharing a low-pass filter among different branches and leveraging the sampling rate transformation theorem that allows for downsampling before filtering.

Among the various angle measurement algorithms, the sum-difference monopulse (MP) method stands out due to its high accuracy, robustness, and easy applicability, and has been extensively applied in radar systems [18,19,20]. However, there are a limited number of studies exploring its application in the field of sound source localization. To summarize, this paper presents a sound source localization algorithm for detecting faults in power transformers, combining polyphase filters with the MP method, to give a solution for achieving high-precision localization of sound sources in transformers within complex environments. This research on spatial localization technology for abnormal sound sources facilitates the precise localization of zones containing abnormal sound sources within transformers and improves maintenance efficiency by narrowing down the troubleshooting scope.

## 2. Signal-Receiving Model of Microphone Array

**Figure 1:**
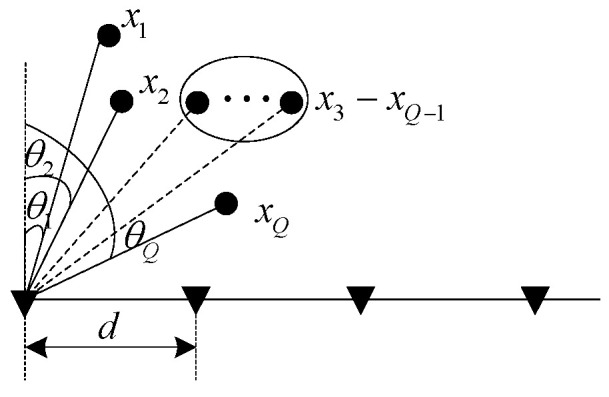
Linear array model of signal receiving.

The model depicted in Figure 1 illustrates the signal received by microphones in a uniform linear array with M elements. In this model, the normal direction represents the direction perpendicular to the array. On the assumption of a far-field model, signals from different sound sources propagate as plane waves coming from various incidence directions. Considering the leftmost signal as the reference element, the received signals of the array can be expressed as [21]

(1) X=a1x1+…+aQxQ+N=Re(∑n=1Qanxn+N)

(2) $$a_{n}={\left[1\ldots \exp(\frac{j2\pi f_{n}(M-1)d\sin\theta_{n}}{c})\right]}^{T}$$

where *d* denotes the element spacing; Q denotes the number of sound sources; x(n) denotes the complex amplitude of the *n*th sound signal; $f_{n}$ denotes the frequency of the *n*th sound signal; $\theta_{n}$ denotes the angle of incidence of the *n*th sound signal; *c* denotes the velocity of sound; *N* denotes the M × 1-dimensional noise vector; $a_{n}$ denotes the steering vector of the array response; Re(∙) denotes the operation to extract the real part; and T denotes the transpose operator.

## 3. Multi-Source Separation Using Polyphase Filter

The sound emitted by running transformers primarily originates from the vibrations of various components, such as windings, iron cores, cooling fans, and others. These components generate vibrations at different frequencies. To localize fault sound sources separately and eliminate noise interference in complex multi-source environments, an analysis filter bank is employed to divide the sound signals received from the transformers into multiple subbands, allowing for the separation of fault sound sources.

Traditional analysis filter banks typically first divide the wideband signals $x_{n}$ into *K* subbands using a modulation process and then shift these subbands to the baseband while decimating them to reduce the sampling rate. The theoretical formulas can be found in [22]. This process necessitates numerous unnecessary calculations, leading to a waste of hardware resources. On the contrary, analysis filter banks based on a polyphase structure share a low-pass filter among branches to improve resource utilization.

An *N*-point FIR filter system function is assumed as

(3) $$H(z)=\sum_{l=0}^{N-1}h(n)$$

Taking *N* as a multiple of *K* and letting *L* = *N*/*K*, we obtain

(4) $$E_{l}(Z^{K})=\sum_{n=0}^{L-1}h(nK+l){\left(z^{K}\right)}^{-n}$$

as the polyphase component of the filter, and then the filter system function can be expressed as

(5) $$H(z)=\sum_{l=0}^{M-1}z^{-l}E_{l}(z^{K})$$

For traditional analysis filter banks, the *k*th (*k* = 0, 1, ..., *K* − 1) output can be expressed as

(6) $$v_{k}(n)=[x(n)*h_{k}(n)]e^{-jw_{k}n}$$

(7) $$y_{k}(m)=v_{k}(n){|}_{n=Km}=\sum_{i=0}^{N-1}x(Km-i)[h_{0}(i)e^{j\omega_{k}(i-Km)}]$$

where x(n) denotes the input signal, $h_{k}(n)$ denotes the *k*th filter response, with $h_{k}(n)=h_{0}(n)e^{j\omega_{k}n}$ , and $h_{0}(n)$ denotes the low-pass prototype filter.

Letting

(8) i=lK+r

Equation (7) can be expressed as

(9) $$y_{k}(m)=\sum_{r=0}^{K-1}e^{j\omega_{k}r}\sum_{l=0}^{L-1}x_{r}(m-l)e_{r}(l)e^{j\omega_{k}K(l-m)}$$

In Equation (9),

(10) $$x_{r}(m-l)=x(Km-Kl-r)$$

(11) $$e_{r}(l)=h_{0}(Kl+r)$$

In odd-channel layout scenarios, the center frequency of the *k*th subband is expressed as

(12) $$w_{k}=-\pi +\frac{2\pi k}{K}$$

Substituting it into Equation (9), we obtain

(13) $$y_{k}(m)=K(IDFT[(x_{r}(m)*e_{r}(m)){\left(-1\right)}^{r}])$$

**Figure 2:**
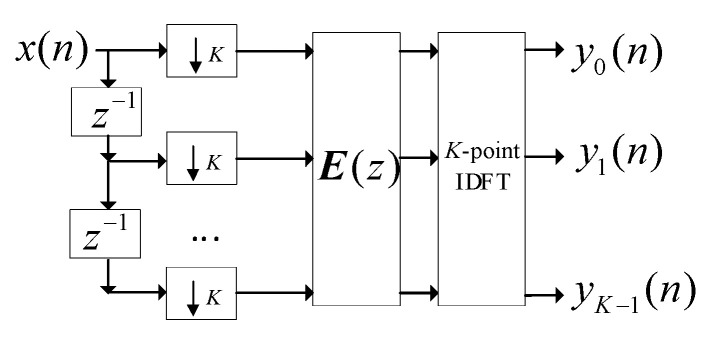
Channelized polyphase filter.

Figure 2 illustrates a channelized polyphase filter. In the polyphase filter (PF) approach, it is important to note that the sequence length of all subbands is 1/*K* of the original sequence length. Specifically, the *0*th and (*K*/2)th subbands carry real signals, while the remaining subbands carry complex signals. Additionally, the information in the 1st to (*K*/2 − 1)th subbands is the same as that in the (*K*/2 + 1)th to (*K* – 1)th subbands. When using PFs for signals received by each element, the output in the *k*th channel of the array is expressed as

(14) $$Y_{k}(m)=a*(K(IDFT[(x_{r}(m)*e_{r}(m){\left(-1\right)}^{r})]))$$

where a denotes the steering vector of the array.

The incorporation of polyphase filters allows for the separation of multiple fault sound sources. Moreover, the sequence length of the subbands containing fault sound sources is reduced to 1/*K* of the original length by downsampling before filtering, which helps reduce the computational workload of subsequent operations, contributing to enhanced calculation efficiency. In addition, sharing a low-pass filter among the polyphase filter branches can greatly improve the utilization rate of system resources.

## 4. Multi-Source Localization Based on Sum-Difference Monopulse in Frequency Domain

### 4.1. Principle of Sum-Difference Monopulse Angle Measurement

Current sound source localization algorithms based on microphone arrays often demand extensive calculations to achieve high localization accuracy, which imposes a high cost for real-world implementation. Therefore, the exploration of a microphone array-based sound source localization technology that combines both accuracy and simplicity holds paramount significance in engineering practice. Considering the extensive applications of the sum-difference MP angle measurement in radar systems, which boasts a simple structure, low calculation complexity, and high accuracy, this section presents a multi-source localization algorithm based on microphone arrays, which aims to strike a balance between accuracy and simplicity, emphasizing the integration of the sum-difference MP technique with the previously mentioned PF in the microphone array.

The sum-difference monopulse angle measurement is achieved by aligning the main lobe of the sum beam with the desired direction and forming the null of the difference beam in the desired direction. We adjust the distance between the microphone array and the transformer and weight the amplitude of each subband to ensure that the transformer’s sound surface is located within the main lobe of the beam. Through the output ratio calculation between the sum and difference beams, a specific value is derived. This value represents the extent of deviation of the beams in the target direction from the beam center, leading to a precise estimation of the target location. Detailed theoretical derivations can be found in reference [23].

Assuming the element spacing of an *M*-element uniform linear array as *d* and the beam pointing as $\theta_{0}$ , the sum beam weight $w_{\Sigma }$ can be taken as

(15) $$w_{\Sigma }=a(\theta_{0})$$

Based on the symmetry of uniform linear arrays, the difference in beam weight $w_{\Delta }$ can be taken as

(16) $$w_{\Delta }={\left[\overset{M/2}{\overset{︷}{-1,\ldots ,-1}},\overset{M/2}{\overset{︷}{1,\ldots ,1}}\right]}^{T}⊙a(\theta_{0})$$

In Equation (16), ⊙ represents the Hadamard product. Then, the outputs of the sum and difference beams in the *k*th subband can be expressed by Equations (17) and (18), respectively.

(17) $$\Sigma (\theta )=w_{\Sigma }^{H}Y_{k}(m)$$

(18) $$\Delta (\theta )=w_{\Delta }^{H}Y_{k}(m)$$

The in-band signaling angle can be estimated from the outputs of the sum and difference beams as follows:

(19) θ∧=θ0+1k′Re(Δ(θ)Σ(θ))

In Equation (19), $k^{\prime }$ denotes the monopulse ratio slope [24,25].

(20) $$k^{\prime }=j\frac{\pi Md}{2\lambda }$$

It can be seen from Equation (20) that the value of $k^{\prime }$ is determined solely by the number of elements (*M*), element spacing (*d*), and signal wavelength ( λ ). The angle measurement accuracy is correlated with the value of $k^{\prime }$ and SNR. Notably, higher values of $k^{\prime }$ and SNR contribute to improved accuracy in angle measurement.

### 4.2. Multi-Source Localization in Subbands

To enhance the accuracy of sound source localization, the complex variational mode decomposition (CVMD) [26] method is used to suppress noise. CVMD is applied within the subbands that contain faulty sound sources. It enables the transformation of the outputs of the sum and difference beams to the frequency domain, thereby providing an enhanced SNR.

(21) $$\hat{\sum }(\theta )=\mathrm{FFT}(w_{\Sigma }^{H}Y_{k}(m))$$

(22) $$\hat{\Delta }(\theta )=\mathrm{FFT}(w_{\Delta }^{H}Y_{k}(m))$$

where FFT denotes the Fast Fourier Transform, and $\hat{\sum }(\theta )$ and $\hat{\Delta }(\theta )$ represent the frequency-domain outputs of $\hat{\sum }(\theta )$ and $\hat{\Delta }(\theta )$ , respectively.

**Figure 3:**
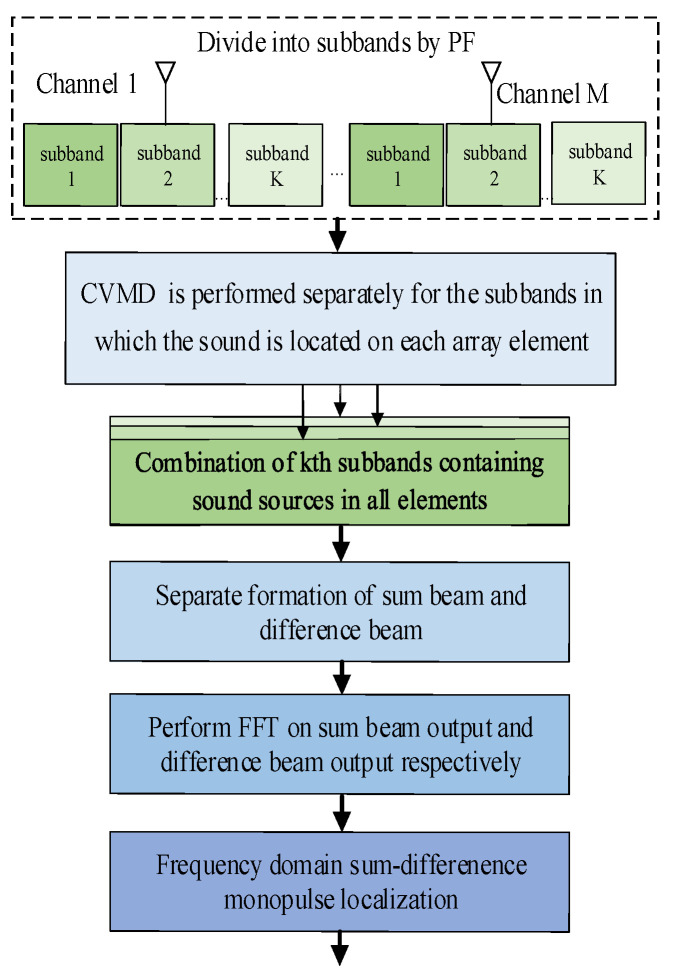
Flow chart of the algorithm for multi-sound source localization.

In conclusion, the proposed algorithm can be explained using a flow chart, as shown in Figure 3. This algorithm, integrating PF and frequency-domain MP angle measurement, can be used for multi-source sound localization of faults in power transformers.

## 5. Analysis of Algorithm Performance

**Figure 4:**
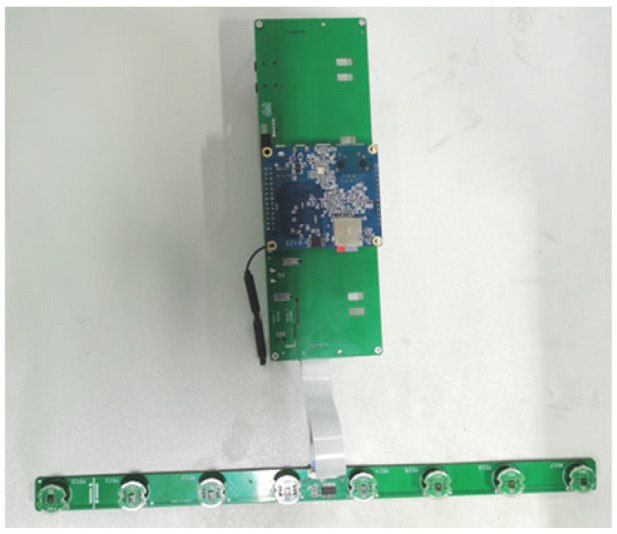
Microphone array.

**Figure 5:**
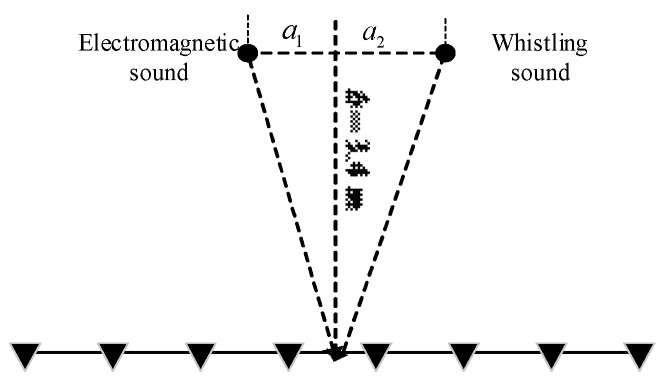
Diagram for experimental data collection.

The performance of the algorithm was verified using the experimental data, which are detailed below. The microphone array used in the experiment was an eight-element line array, with each array element having a center spacing of 4.25 cm and a sampling rate of 96 KHz. Figure 4 shows the microphone array used in the experiment. The first transformer sound source to be estimated emitted electromagnetic sound, while the second sound source emitted a whistling sound. Figure 5 shows a sketch of the experimental setup. The system parameters are shown in Table 1.

**Figure 6:**
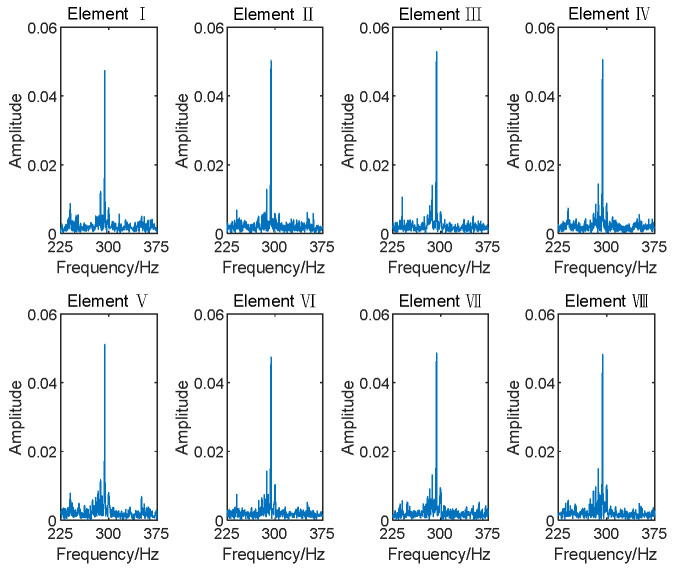
Subband spectrum of array elements for electromagnetic sound.

**Figure 7:**
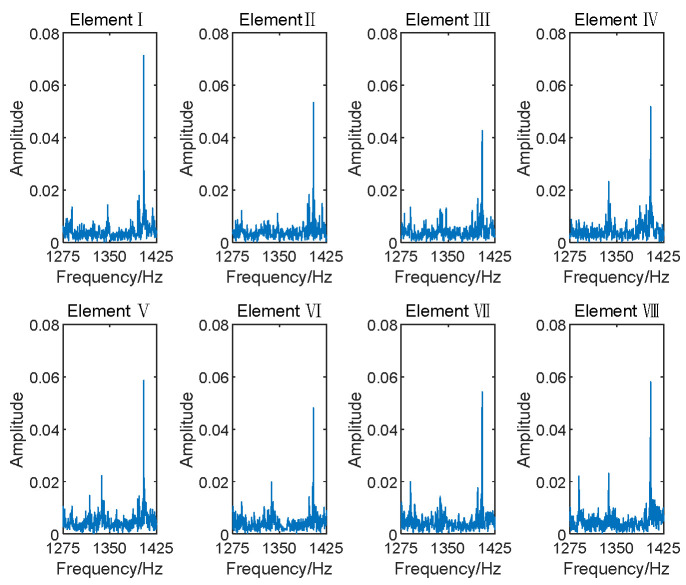
Subband spectrum of array elements for whistling sound.

The entire Nyquist spectrum was divided into 640 subbands using PFs, with each subband having a bandwidth of 150 Hz. In engineering applications, the transformer’s voiceprint needs to be detected and identified before localization. In fault identification, the frequency domain information corresponding to different faulty voiceprints can be determined, and this can be used to determine the subbands in which the faulty voiceprint is located. The electromagnetic sound was localized in the 323rd subband, while the whistling sound was in the 330th subband. Figure 6 and Figure 7 illustrate the frequency spectrograms of the subbands containing these two sound sources in their respective elements.

**Figure 8:**
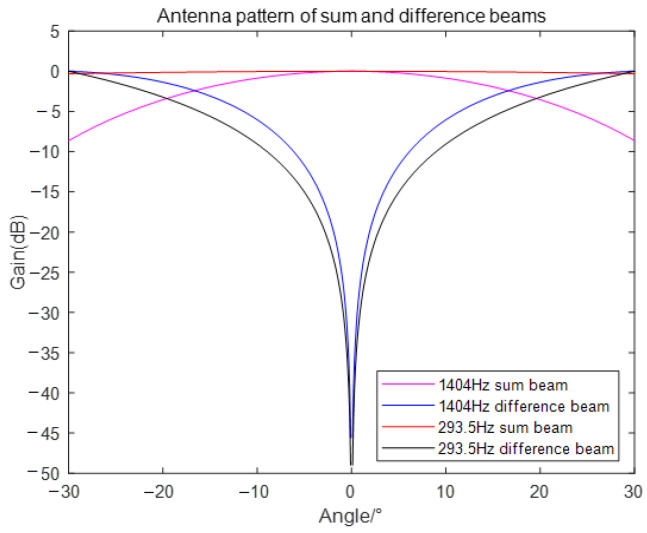
Antenna pattern of sum and difference beams.

**Figure 9:**
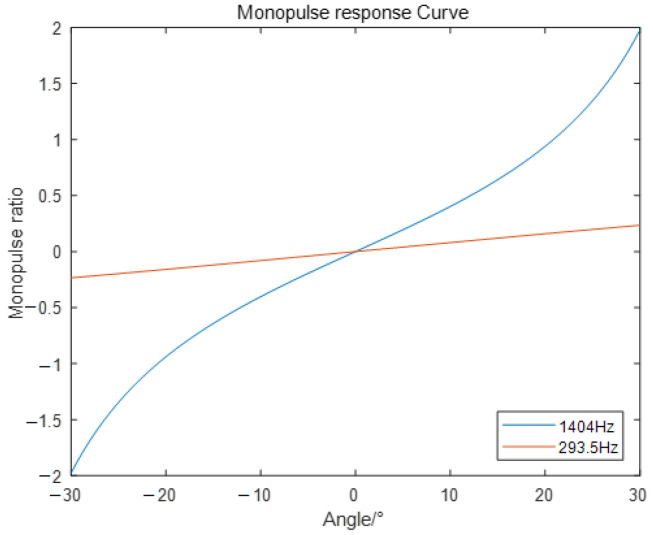
Monopulse response curves.

Figure 8 and Figure 9 show the antenna patterns for the sum and difference beams corresponding to $f_{1}$ and $f_{2}$ , along with their monopulse response curves. These illustrations demonstrate that the main lobe of the beams is aligned at an angle of 0 degrees, indicating a notch in the difference beams. It is important to note that the sum-difference monopulse angle measurement exploits the linear interval of the monopulse response curve near the main lobe. Consequently, as the sound source frequency increases, the main lobe narrows, resulting in a larger value of $k^{\prime }$ and higher accuracy in angle measurement. Typically, the range of the 3dB main lobe attenuation is considered the linear interval for angle measurement. Thus, in Figure 8, the angle measurement range for the sum and difference beams of the electromagnetic sound is approximately −30° to 30°, while for the whistling sound, it is approximately −15° to 15°.

**Figure 10:**
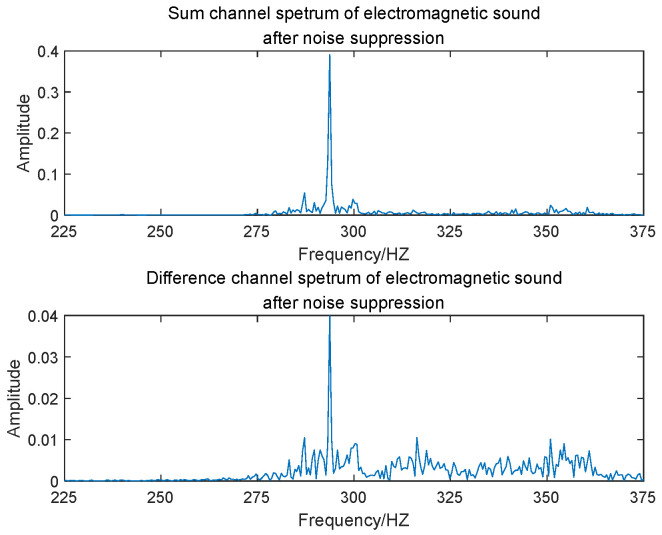
FFT outputs of sum and difference beams of electromagnetic sound.

**Figure 11:**
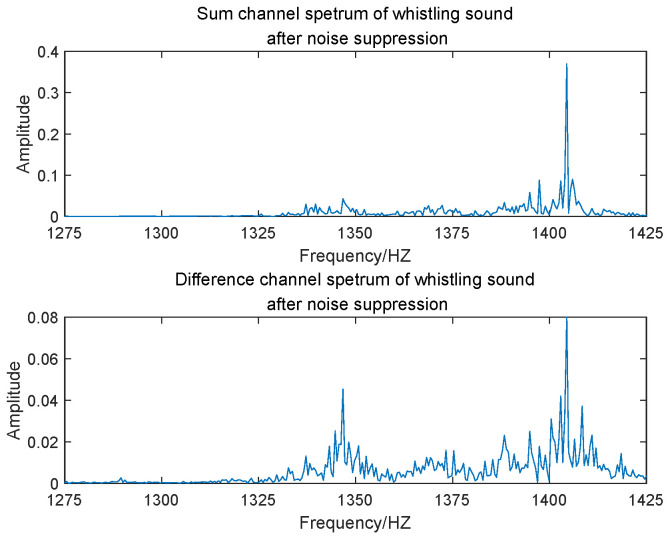
FFT outputs of sum and difference beams of whistling sound.

Figure 10 and Figure 11 show the FFT outputs of the sum and difference beams, respectively, for the electromagnetic sound and the whistling sound. In the validation of the actual measurement data, we used two different spectra of electromagnetic and whistling sounds corresponding to the two kinds of faulty sound patterns for localization. Table 2 shows the offsets in the conversion results obtained from angle estimation using MP and MUSIC algorithms. Through a comparison of these results, it can be concluded that the algorithm proposed in this paper demonstrates significantly improved localization accuracy, both at high and low frequencies.

## 6. Conclusions

To tackle the challenges faced by classical microphone array-based sound source localization algorithms, which often struggle to balance accuracy and simplicity and, in particular, encounter significant performance degradation when addressing transformer faults in environments with multiple sound sources, this paper investigates the algorithm for multi-sound source localization of power transformer faults based on polyphase filters. The algorithm incorporates PFs to first divide the received signal into different subbands, enabling the downsampling of signals in the time-domain subbands and the separate formation of sum and difference beams. The outputs of these sum and difference beams are transformed into the frequency domain, and their ratio is utilized to achieve high-precision localization of fault sound sources simultaneously. In this paper, the performance of the proposed algorithm is evaluated by comparing its angle measurement results with those obtained using the MUSIC algorithm. The experimental data demonstrate that the proposed algorithm outperforms the MUSIC algorithm in terms of localization accuracy when faced with array amplitude and phase errors. Moreover, the proposed algorithm offers practical advantages due to its lower computational complexity, making it suitable for real-world applications. This algorithm has proven effective in achieving high-precision localization of sound sources for transformer faults. It helps narrow down the troubleshooting scope and assists operation and inspection personnel in targeting fault sources when abnormalities initially emerge. These benefits significantly enhance the efficiency of operation and inspection, thereby demonstrating substantial application potential. The present study also establishes a strong foundation for future investigations concentrating on ways to further suppress interference and enhance the localization accuracy of this algorithm in complex environments where interference and noise coexist.

## Figures and Tables

**Table 1:** Parameters of experimental system.

Parameter	Symbol	Value
Sampling rate	$f_{s}$	96,000 Hz
Number of elements	M	8
Element spacing	d	0.0425 m
Center frequency of electromagnetic sound	$f_{1}$	293.5 Hz
Center frequency of whistling sound	$f_{2}$	1404 Hz
True offset value of electromagnetic sound	$a_{1}$	−0.3 m
True offset value of whistling sound	$a_{2}$	0.36 m
Velocity of sound	c	340 m/s
Number of subbands	K	data

**Table 2:** Angle measurement results of different algorithms.

Algorithm	$f_{1}$ Estimation Offset/m	$f_{1}$ Estimation Error/m	$f_{2}$ Estimation Offset/m	$f_{2}$ Estimation Error/m
MP	−0.6039	−0.3039	0.3322	−0.0278
MUSIC	−0.6850	−0.3850	0.2601	−0.0999

## Data Availability

The data used to support the findings of this study are available from the corresponding author upon request.
